# Serum uric acid and risk of incident chronic kidney disease: a national cohort study and updated meta-analysis

**DOI:** 10.1186/s12986-021-00618-4

**Published:** 2021-10-19

**Authors:** Nianwei Wu, Jing Xia, Sen Chen, Chuan Yu, Ying Xu, Chengfu Xu, Tong Yan, Ningxiu Li, Yanjun Liu, Xiong-Fei Pan

**Affiliations:** 1 Department of Health and Social Behavior, West China School of Public Health and West China Fourth Hospital, Sichuan University, Chengdu, Sichuan China; 2 Kidney Disease Center, The First Affiliated Hospital, College of Medicine, Zhejiang University, Hangzhou, Zhejiang China; 3 Department of Gastroenterology, The First Affiliated Hospital, College of Medicine, Zhejiang University, Hangzhou, Zhejiang China; 4 Center for Obesity and Metabolic Health, The Third People’s Hospital of Chengdu & The Affiliated Hospital of Southwest Jiaotong University, Chengdu, Sichuan China; 5 Center of Gastrointestinal and Minimally Invasive Surgery, Department of General Surgery, The Third People’s Hospital of Chengdu & The Affiliated Hospital of Southwest Jiaotong University, Chengdu, Sichuan China; 6 Division of Epidemiology, Department of Medicine, Vanderbilt Epidemiology Center, Vanderbilt University Medical Center, Nashville, TN USA; 7 Department of Epidemiology and Biostatistics, and Ministry of Education Key Lab of Environment and Health, School of Public Health, Tongji Medical College, Huazhong University of Science and Technology, Wuhan, Hubei China; 8 The George Institute for Global Health, Faculty of Medicine, University of New South Wales, Sydney, NSW Australia

**Keywords:** Serum uric acid, Chronic kidney disease, Chinese, Cohort study, Meta-analysis

## Abstract

**Background:**

We prospectively examined the association between serum uric acid (SUA) levels and chronic kidney disease (CKD) in China and updated the evidence through a comprehensive meta-analysis of prospective studies worldwide.

**Methods:**

Our original analyses were based on data from the China Health and Retirement Longitudinal Study. The primary exposure of interest was SUA at baseline, and the main outcome was incident CKD. Logistic regression models were used to examine the association between SUA levels and incident CKD. A meta-analysis was performed to pool our effect estimate and those from other cohort studies.

**Results:**

During a 4-year follow-up, 180 participants developed incident CKD. Participants in the highest SUA quartile were 2.73 times as likely to develop incident CKD compared to those in the lowest quartile (multivariable-adjusted OR, 2.73; 95% CI, 1.65–4.50). Each 1 mg/dL increment in the SUA levels was associated with a 49% increased risk of incident CKD (multivariable-adjusted OR, 1.49; 95% CI, 1.28–1.74). In the meta-analysis of 30 cohort studies (including the current study), pooled relative risks (95% CIs) of incident CKD were 1.15 (1.10–1.21) for SUA each 1 mg/dL increment, 1.22 (1.14–1.30) for the highest versus lowest SUA group, and 1.17 (1.12–1.23) for hyperuricemia versus no hyperuricemia.

**Conclusions:**

Baseline SUA levels were associated with higher risk of incident CKD in middle-aged and elderly Chinese adults, and this positive association was confirmed in the meta-analysis of multiple cohort studies. Our findings may imply that SUA levels need to be routinely monitored for future CKD risk.

**Supplementary Information:**

The online version contains supplementary material available at 10.1186/s12986-021-00618-4.

## Background

Chronic kidney disease (CKD) is a global public health challenge. The age-standardized prevalence rates of CKD were 8.7% worldwide and 7.2% in China in 2017 [1]. CKD is strongly associated with development of end-stage renal disease, cardiovascular events, and premature mortality [2, 3]. In addition, it compromises health-related quality of life and causes substantial financial burden for the patients [3]. It is thus essential to investigate potential modifiable risk factors and early interventions for CKD.

Serum uric acid (SUA), an end-product of the metabolism of purine nucleotides, is commonly elevated in patients with CKD [4]. Although high SUA levels could be due to impaired clearance of uric acid in CKD, it is long speculated that high SUA levels may be implicated in the development and progression of CKD [5]. A systematic review of 15 cohort studies among 99,205 participants and 3,492 incident CKD cases showed a risk increase of 22% for each increment of 1 mg/dL SUA [6]. Of these included studies, four studies were conducted in China: three in health care settings in Chinese Taiwan [7–9] and one in a community setting in Chinese mainland [10]. Besides the restricted health care settings that may affect the generalizability, results from these studies were not very consistent. For example, of the four studies in Chinese populations, two in health care settings reported borderline or null associations of CKD with each unit of SUA increase in Chinese Taiwan [7, 9]. In addition, the positive association between SUA levels and CKD seemed to be stronger in Asians versus non-Asians, and in health-check populations versus non-health-check populations, despite a lack of statistical significance for the heterogeneity [6]. In this context, we hypothesized that SUA could be a risk factor for CKD in Chinese and proposed to test this hypothesis in a population-based cohort study with a large sample size in mainland China.

To this end, we conducted analyses using data from the China Health and Retirement Longitudinal Study (CHARLS), a national population-based longitudinal study, to assess the association between baseline SUA levels and risk of incident CKD. In addition, we conducted a comprehensive meta-analysis of findings from literature to corroborate our work in the Chinese population and update the evidence on this topic.

## Methods

### Study design

The CHARLS is an ongoing nationally representative study among adults aged 45 years or older that aims to assess aging-related issues in China. Details of the study design, survey methods, and procedures were described elsewhere [11]. Briefly, a multi-stage probability sampling procedure was performed to recruit 17,708 participants from 450 communities of 28 provinces at baseline (2011–2012). Trained staff collected data using face-to-face questionnaires via computer-assisted personal interviews, physical measurements, and blood samples for biomarker measurements at baseline and during the follow-up until 2015–2016. While the original CHARLS did not specifically address the etiology of kidney diseases, we utilized data on SUA and covariates at baseline and CKD outcomes during the follow-up to examine the association of SUA and risk of incident CKD. The original CHARLS was approved by the Biomedical Ethics Review Committee of Peking University (IRB00001052-11,015), and all participants provided informed consent.

The eligibility of study participants and the study flow are presented in Additional file 1: Figure S1. Of the 17,708 study participants in CHARLS, we excluded 7,081 participants without blood samples and 496 participants who did not complete questionnaires at baseline. Of the remaining 10,131 participants, we excluded the participants who lacked information on SUA (n = 163) and CKD (n = 247) at baseline.

We also excluded the participants whose baseline estimated glomerular filtration rate (eGFR) was < 60 mL/min/1.73m^2^ (n = 114), who used medications for kidney diseases (n = 313), or who had prevalent malignancies (n = 51), cardiovascular diseases (i.e., heart disease and stroke) (n = 1,565). Of the 7,678 eligible participants, we excluded the 2,188 participants who were lost to follow-up and 165 participants who lacked information on CKD in 2015–2016. 779 participants were additionally excluded due to data missing for age (n = 6), sex (n = 6), education level (n = 6), residence (n = 7), alcohol consumption (n = 47), body mass index (BMI, n = 679), and systolic blood pressure (BP; n = 28) at baseline. 4,546 participants were included in final analysis. The 3,132 eligible participants who were excluded were more likely to be older, from urban areas, and to suffer from hypertension or diabetes, but were less likely to be never smokers, compared to the included 4,546 participants (P ≤ 0.020 for all, Additional file 1: Table S1).

### Study exposure and outcome

The primary exposure was SUA at baseline, which was measured in the Youanmen Center for Clinical Laboratory of Capital Medical University using the SUA Plus method. We categorized SUA levels into quartiles (< 3.5, 3.6–4.2, 4.3–5.0, and > 5.1 mg/dL). Hyperuricemia was defined as a level of SUA ≥ 7.0 mg/dL in men or ≥ 6.0 mg/dL in women [12].

The research outcome, incident CKD, was primarily defined as eGFR < 60 mL/min/1.73 m^2^ in 2015–2016. The eGFR was calculated using the Chronic Kidney Disease Epidemiology Collaboration (CKD-EPI) Study equation: GFR = $141 \times min({\text{Scr}}/\kappa ,{ }1)^{\alpha } \times {\text{max}}\left( {{\text{Scr}}/\kappa ,1} \right)^{ - 1.209} \times 0.993^{{{\text{Age}}}} \times 1.108\left[ {\text{if female}} \right] \times 1.159\left[ {\text{if black}} \right],$ where Scr is serum creatinine, $\kappa$ is 0.7 for females and 0.9 for males, $\alpha$ is − 0.329 for females and − 0.411 for males, min means the minimum of ${\text{Scr}}/\kappa$ or 1, and max means the maximum of ${\text{Scr}}/\kappa$ or 1 [13]. In a sensitivity analysis, we used the Modification of Diet in Renal Disease (MDRD) Study equation [14] to alternatively estimate eGFR: eGFR = $175 \times \left( {Scr} \right)^{ - 1.154} \times \left( {age} \right)^{ - 0.203} \times 0.742\left[ {\text{if female}} \right] \times 1.212\left[ {\text{if black}} \right],$ where Scr is serum creatinine. Serum creatinine was measured by the rate-blanked and compensated Jaffe creatinine method.

### Covariate measurements

Major covariates including sociodemographic characteristics, health-related lifestyle and behaviors, medical conditions, and anthropometrics were collected at baseline (2011–2012). Sociodemographic characteristics comprised age, sex (men and women), educational level (illiterate, primary school, and middle school or above) and residence (rural and urban). Health-related lifestyle and behaviors comprised cigarette smoking (current, former, and never), and alcohol drinking (current, former, and never). Current smoking was defined as having the habit of smoking cigarettes, cigars, tobacco, or pipe currently, and current drinking was defined as having drunk any alcoholic beverages (wine, beer, or liquor) for more than once a month in the past year. Medical conditions included prevalent hypertension, diabetes mellitus, and dyslipidemia (yes or no). Body weight and height were measured using a digital weight scale and stadiometer. BMI was defined as the body weight in kilograms divided by the square of the height in meters (kg/m^2^). BP was measured three times using an electronic blood pressure monitor (Omron HEM-7112; Omron [Dalian] Co, Dalian, China) on the right upper arm after 5 min of seated rest. Blood samples were collected from study participants by trained staff based on a standard protocol for biomarker measurements. Fasting blood glucose (FBG), triglyceride (TG), high-density lipoprotein cholesterol (HDL-C), low-density lipoprotein cholesterol (LDL-C), and total cholesterol (TC) were measured by the enzymatic colorimetric test. Glycosylated hemoglobin (HbA1c) was measured through the high performance liquid chromatography. Hypertension was defined as systolic BP ≥ 140 mmHg or diastolic BP ≥ 90 mmHg or self-reported physician diagnosis [15]. Prevalent diabetes was determined based on fasting blood glucose ≥ 126 mg/dL, HbA1c ≥ 6.5%, or self-reported physician diagnosis [16]. Dyslipidemia was defined as either or a combination of TC ≥ 240 mg/dL, LDL-C ≥ 160 mg/dL, HDL-C < 40 mg/dL, TG ≥ 200 mg/dL, or self-reported physician diagnosis, according to the 2007 Chinese guidelines on the prevention and treatment of dyslipidemia [17].

### Statistical analyses

Continuous variables were summarized as mean and standard deviation (SD), while categorical variables were presented as frequency and percentage. Baseline characteristics were compared using ANOVA (for continuous variables) or Pearson’s chi-square (for categorical variables) tests across SUA quartiles.

Logistic regression models were employed to assess the relationship between baseline SUA and incident CKD with adjustments for a priori identified covariates in a stepwise manner. Odds ratios (ORs) with corresponding 95% CIs were estimated. In Model 1, we adjusted for age (continuous, years), sex (male and female), residence area (rural and urban), and education level (illiterate, primary school, and middle school or above). In Model 2, we adjusted for variables in Model 1 plus BMI (continuous, kg/m^2^), smoking status (never, former, and current), and alcohol consumption (never, former, and current). In Model 3, we adjusted for variables in Model 2 plus systolic BP (continuous, mmHg), TC (continuous, mg/dL), TG (continuous, mg/dL), and prevalent diabetes mellitus (yes and no). In all these models, SUA was assessed as either a categorical variable (dichotomous or in quartiles) or a continuous variable (each 1 mg/dL). Linear trends across SUA quartile groups were estimated by modeling SUA as the median of each quartile. In addition, subgroup analyses were conducted by sex (men and women), age (< 60 and ≥ 60 years), education level (illiterate, primary school, and middle school or above), residence (rural and urban), BMI (< 24, 24.0–27.9, and ≥ 28 kg/m^2^ based on the Chinese criteria for overweight and obesity) [18], smoking status (current, former, and never), alcohol consumption (current, former, and never), hypertension (yes and no), prevalent diabetes mellitus (yes and no), and dyslipidemia (yes and no), and potential effect modifications (interactions) were explored by adding a product term of the stratifying variable and SUA to the final model and examined using a likelihood ratio test.

Four sensitivity analyses were conducted. First, we adjusted for prevalent dyslipidemia (yes and no), and hypertension (yes and no) as binary variables instead of continuous variables in the final model. Second, we conducted multiple imputations for 779 participants with missing covariates followed by logistic regression modeling. Under the assumption that data were missing at random, missing values were replaced by imputed ones from ten duplicate datasets that were created to reduce sampling variability from the imputation simulation, and estimates from the ten imputed datasets were combined to obtain the effect estimates and 95% CI. Third, eGFR was estimated using the MDRD Study equation prior to CKD ascertainment. Fourth, we estimated the time-mean SUA as the arithmetic mean of the baseline (2011–2012) and follow-up (2015–2016) measurements, and modelled this variable as in the main analyses. All analyses were conducted using Stata 16.0 (Stata Corp LLC, College Station, Texas, US). *P* < 0.05 was defined as statistical significance.

### Meta-analysis of cohort studies

We conducted a meta-analysis of data from our study and other cohort studies that assessed the relationship between the SUA levels and incident CKD in adults. A literature search was performed using Medline (1948 to present) and Embase (1974 to present) for published studies up to July 30, 2021. We combined MeSH terms and keywords for “uric acid”, “hyperuricemia”, “chronic kidney disease”, and “chronic renal insufficiency” in our search strategy. Records were screened by two investigators (NW and JX) independently with the following eligibility criteria: (1) cohort studies; (2) assessment of the association between SUA and new-onset CKD; and (3) reporting effect estimates and corresponding 95% CIs such as hazard ratio (HR) or OR. Study-specific effect estimates were pooled using a random-effects model (Q test’s *P* < 0.01) or fixed-effects model (when Q test’s *P* > 0.01). When studies reported effect estimates for men and women separately, they were pooled in individual studies by using random-effects meta-analysis, which were used to combine with those in other studies for the final meta-analysis. If effect estimates were reported as ORs, they were converted to relative risks (RRs) using the formula (RR = OR/([1 − pRef] + [pRef × OR]), where pRef is the prevalence of the outcome in the reference group. HRs were regarded as approximate RRs for the meta-analysis. Subgroup analyses were conducted by major study characteristics including sex (men and women), mean age (< 60 and ≥ 60 years), population (Asian and non-Asian), and average follow-up (< 4.6 and ≥ 4.6 years, using the median of average follow-up durations across studies as the cut-off). Meta-analyses were conducted using Stata 16.0 (Stata Corp LLC, College Station, Texas, US). *P* < 0.05 was defined as statistical significance.

## Results

### Baseline characteristics

Of 4,546 participants, the mean age was 58.57 (SD, 8.67) years, and 2,099 (46.2%) were men (Table 1). Baseline mean (SD) statistics were 4.34 (1.20) mg/dL for SUA, 0.76 (0.16) mg/dL for serum creatinine, and 94.15 (12.53) mL/min per 1.73 m^2^ for eGFR. Participants with higher SUA were more likely to be older, men, urban residents, current/former smokers, or current alcohol drinkers, and to have hypertension and dyslipidemia. In addition, BMI, systolic BP, diastolic BP, TG, TC and serum creatinine levels increased with SUA levels, while HDL cholesterol decreased with SUA levels.

**Table 1:** Baseline characteristics of participants across SUA quartiles (n = 4,546)

Characteristics			SUA (mg/dL)			*P*-value*
	Total	Quartile 1	Quartile 2	Quartile 3	Quartile 4	
		(< 3.5)	(3.6–4.2)	(4.3–5.0)	(> 5.1)	
Men, n (%)	2099 (46.2%)	251 (21.5%)	420 (37.1%)	633 (55.0%)	795 (72.4%)	< 0.001
Education level, n (%)						< 0.001
Illiterate	1310 (28.8%)	428 (36.7%)	364 (32.1%)	310 (27.0%)	208 (18.9%)	
Primary school	1882 (41.4%)	438 (37.6%)	468 (41.3%)	459 (39.9%)	517 (47.1%)	
Middle school or above	1354 (29.8%)	299 (25.7%)	301 (18.4%)	381 (33.1%)	373 (34.0%)	
Urban residence, n (%)	626 (13.8%)	135 (11.6%)	145 (12.8%)	160 (13.9%)	186 (16.9%)	0.002
Smoking status, n (%)						< 0.001
Current	1388 (30.5%)	220 (18.9%)	292 (25.8%)	410 (35.7%)	466 (42.4%)	
Former	359 (7.9%)	47 (4.0%)	67 (5.9%)	92 (8.0%)	153 (13.9%)	
Never	2799 (61.6%)	898 (77.1%)	774 (68.3%)	648 (56.3%)	479 (43.6%)	
Alcohol consumption, n (%)						< 0.001
Current	1202 (26.4%)	170 (14.6%)	239 (21.1%)	328 (28.5%)	465 (42.3%)	
Former	368 (8.1%)	77 (6.6%)	94 (8.3%)	109 (9.5%)	88 (8.0%)	
Never	2976 (65.5%)	918 (78.8%)	800 (70.6%)	713 (62.0%)	545 (49.6%)	
Hypertension, n (%)	1171 (25.8%)	224 (19.2%)	255 (22.5%)	319 (27.7%)	373 (34.0%)	< 0.001
Prevalent diabetes mellitus, n (%)	671 (14.8%)	169 (14.5%)	151 (13.3%)	182 (15.8%)	169 (15.4%)	0.348
Dyslipidemia, n (%)	1879 (41.3%)	392 (33.6%)	440 (38.8%)	525 (45.7%)	522 (47.5%)	< 0.001
Age, mean (SD), yrs	58.57 (8.67)	57.28 (8.48)	58.26 (8.40)	58.96 (8.75)	59.84 (8.85)	< 0.001
BMI, mean (SD), kg/m^2^	23.52 (3.83)	23.11 (3.50)	23.40 (3.93)	23.67 (3.79)	23.94 (4.04)	< 0.001
Systolic BP, mean (SD), mmHg	129.10 (21.12)	125.91 (19.62)	127.53 (20.32)	130.25 (22.77)	132.93 (21.00)	< 0.001
Diastolic BP, mean (SD), mmHg	75.31 (11.90)	73.64 (11.05)	74.80 (11.84)	75.90 (12.31)	77.01 (12.12)	< 0.001
Triglycerides, mean (SD), mg/dL	127.80 (91.54)	113.80 (75.23)	121.47 (83.24)	129.72 (93.76)	147.15 (108.29)	< 0.001
Total cholesterol, mean (SD), mg/dL	194.14 (37.34)	189.61 (35.93)	192.91 (36.92)	196.06 (38.28)	198.22 (37.71)	< 0.001
HDL Cholesterol, mean (SD), mg/dL	51.58 (15.18)	52.95 (14.23)	52.03 (14.67)	51.26 (15.88)	50.01 (15.76)	< 0.001
LDL Cholesterol, mean (SD), mg/dL	117.41 (34.35)	115.58 (32.06)	117.34 (32.96)	118.83 (35.08)	117.93 (37.16)	0.135
Fasting glucose, mean (SD), mg/dL	108.76 (32.34)	110.33 (40.72)	108.00 (33.38)	108.18 (27.66)	108.46 (24.90)	0.280
SCr, mean (SD), mg/dL	0.76 (0.16)	0.66 (0.12)	0.72 (0.13)	0.78 (0.14)	0.87 (0.15)	< 0.001
eGFR, mean (SD), mL/min per 1.73 m^2^	94.15 (12.53)	99.57 (10.83)	95.82 (11.16)	92.77 (12.47)	88.14 (12.74)	< 0.001

BMI, body mass index; BP, blood pressure; eGFR, estimated glomerular filtration rate; HDL, high-density lipoprotein; LDL, low-density lipoprotein; SCr, serum creatinine; SUA, serum uric acid; SD, standard deviation; yrs, years

*P value by ANOVA for continuous variables and chi-square test for categorical variables

### Association between SUA levels and risk of incident CKD

During the 4-year follow-up period, 180 participants developed incident CKD (Table 2). Despite a lack of statistical significance, hyperuricemia seemed to be positively associated with incident CKD (multivariable OR, 1.73; 95% CI, 0.98–3.06). Participants in the fourth SUA quartile were 2.73 times as likely to develop incident CKD compared to those in the first quartile (OR, 2.73; 95% CI, 1.65–4.50; Table 2). A linear trend was noted for the association between SUA quartiles and incident CKD (*P* for trend < 0.001). In addition, each 1 mg/dL increment in the SUA levels was associated with a 49% increased risk of incident CKD after adjustment for potential confounders (OR, 1.49; 95% CI, 1.28–1.74) (Table 2). When prevalent dyslipidemia and hypertension were adjusted for instead of continuous measurements or when multiple imputations were employed for variables with data missing, there were no appreciable changes in the effect estimates (Additional file 1: Table S2-3). When we used the MDRD study equation to estimate GFR for CKD ascertainment, elevated SUA levels showed stronger positive associations with risk of incident CKD (OR, 3.18; 95% CI, 2.01–5.03 for Quartile 4 versus 1; OR, 1.52; 95% CI, 1.32–1.75 for each 1 mg/dL increment; Additional file 1: Table S4). In the sensitivity analysis using time-mean SUA as the exposure, effect estimates were stronger than those from the main analyses (OR, 4.16; 95% CI, 2.51–6.90 for Quartile 4 versus 1; OR, 1.97; 95% CI, 1.69–2.30 for each 1 mg/dL increment; Additional file 1: Table S5). Our exploratory subgroup analyses found consistent positive associations across all subgroups (*P* for interaction ≥ 0.167 for all, Additional file 1: Figure S2).

**Table 2:** Association between SUA levels and incident CKD in China

	CKD* / no. of participants	Model 1 OR (95% CI)	Model 2 OR (95% CI)	Model 3 OR (95% CI)
Hyperuricemia^#^				
No	156/4208	1.00 (reference)	1.00 (reference)	1.00 (reference)
Yes	23/338	1.87 (1.07–3.27)	1.82 (1.04–3.20)	1.73 (0.98–3.06)
SUA levels				
Quartile 1	27/1165	1.00	1.00	1.00
Quartile 2	38/1133	1.46 (0.88–2.41)	1.44 (0.87–2.39)	1.44 (0.86–2.38)
Quartile 3	49/1150	1.93 (1.18–3.15)	1.89 (1.16–3.10)	1.89 (1.15–3.10)
Quartile 4	66/1098	2.80 (1.72–4.56)	2.77 (1.69–4.55)	2.73 (1.65–4.50)
*P* for trend		*P* < 0.001	*P* < 0.001	*P* < 0.001
Each 1 mg/dL		1.49 (1.29–1.73)	1.50 (1.29–1.74)	1.49 (1.28–1.74)

Model 1: adjusted for age (continuous, years), sex (male and female), residence area (rural and urban), education level (illiterate, primary school, middle school or above). Model 2: adjusted for variables in Model 1 plus body mass index (continuous, kg/m^2^), smoking status (never, former, and current), and alcohol consumption (never, former, and current); Model 3: adjusted for variables in Model 2 plus systolic BP (continuous, mmHg), total cholesterol (continuous, mg/dL), triglyceride (continuous, mg/dL), and prevalent diabetes mellitus (yes and no)

CKD, chronic kidney disease; CI, confidence interval; OR, odds ratio; SUA, serum uric acid

^*^CKD which was defined as eGFR < 60 mL/min/1.73 m^2^ based on the Chronic Kidney Disease Epidemiology Collaboration (CKD-EPI)

^#^ Hyperuricemia was defined as a level of SUA ≥ 7.0 mg/dL in men or ≥ 6.0 mg/dL in women

### Meta-analysis for associations between SUA levels and incident CKD

**Fig. 1:**
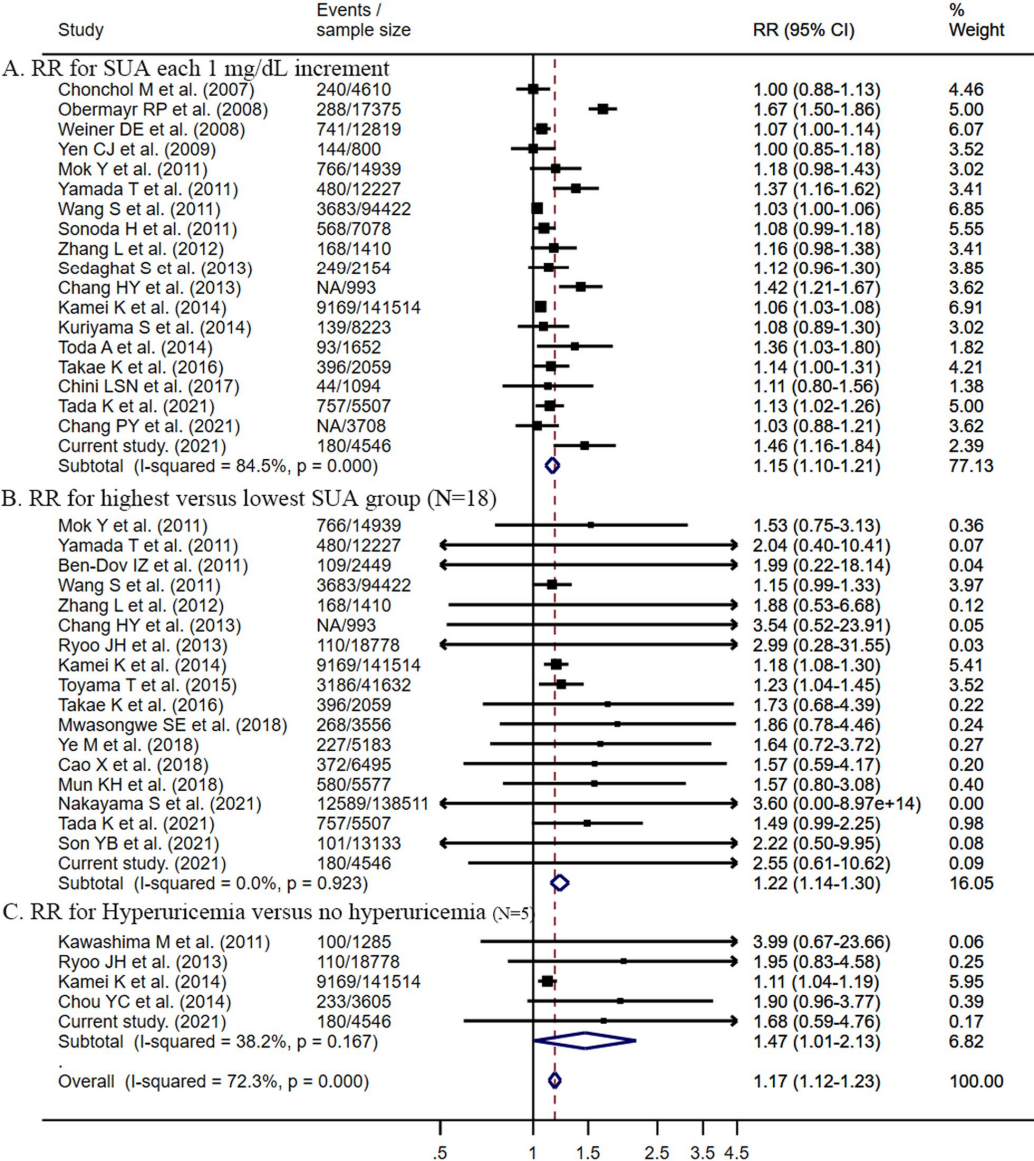
Meta-analysis of the association between SUA and incident CKD

**Fig. 2:**
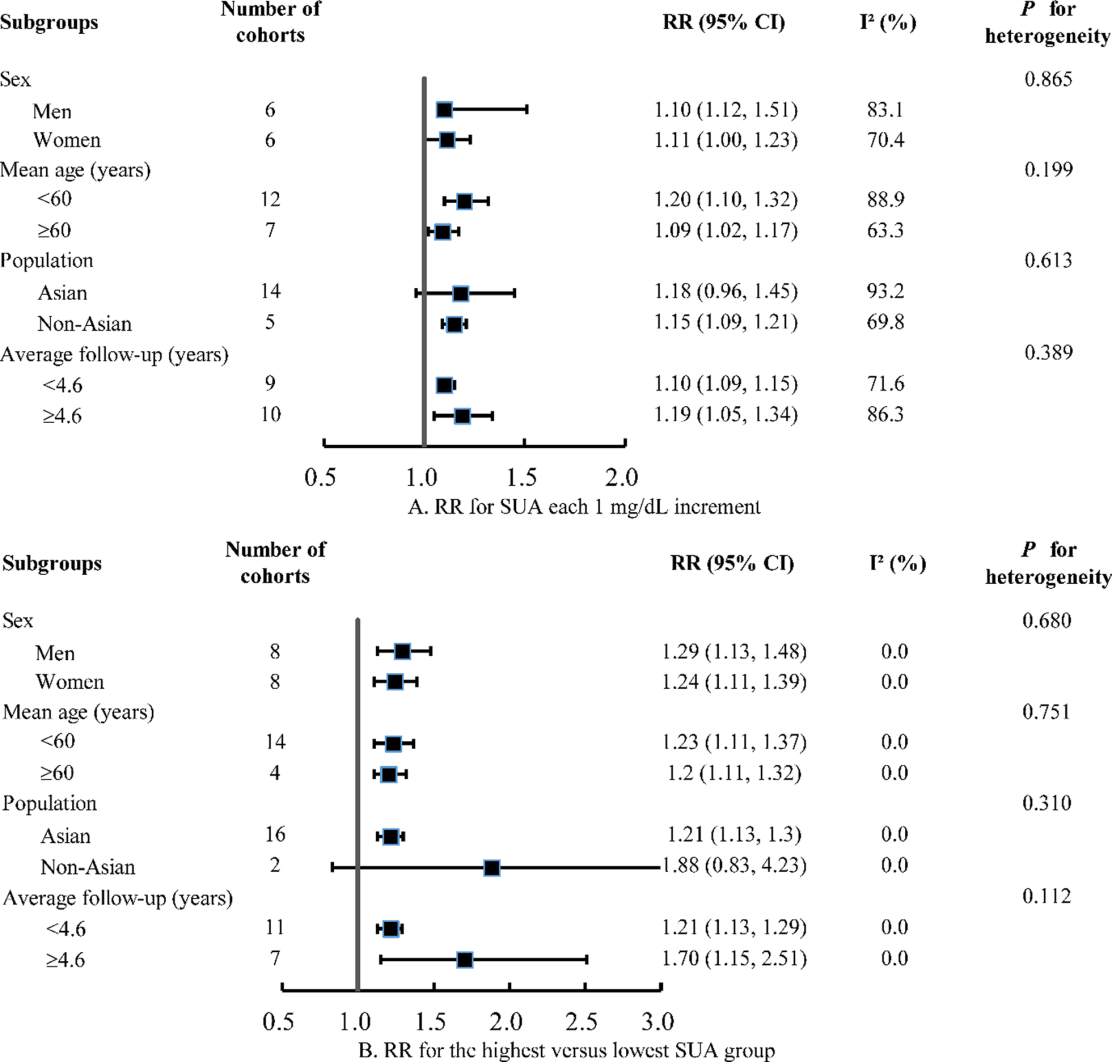
Subgroup analyses for the association between SUA and incident CKD

A total of 6,135 records were retrieved from two databases. After the exclusion of records that did not meet eligibility criteria (Additional file 1: Figure S3), a total of 30 studies (including the current study) were included in the meta-analysis (Additional file 1: Table S6). These studies comprised 577,334 participants and 35,980 incident cases of CKD (average follow-up durations: 4.6 years). 19 studies reported effect estimates for SUA as a continuous variable, 18 studies for SUA in quartiles, and 5 studies for SUA as a dichotomous variable. Pooled RRs (95% CIs) in relation to incident CKD were 1.15 (1.10–1.21) for SUA each 1 mg/dL increment, 1.22 (1.14–1.30) for the fourth versus first SUA quartile, and 1.17 (1.12–1.23) for hyperuricemia versus no hyperuricemia (Fig. 1). We found no evidence of heterogeneity in subgroups by sex, age, population, and follow-up duration (*P* ≥ 0.112 for all; Fig. 2).

## Discussion

In this national population-based cohort study in China, we found that higher SUA levels were associated with an increased risk of incident CKD, independent of other risk factors and consistent across subgroups. This positive association was also confirmed by findings in a meta-analysis of 30 cohort studies in multiple populations.

In our study, participants with each 1 mg/dL increment in the SUA levels had 49% higher risk of incident CKD during the four-year follow-up, and participants in the fourth SUA quartile were 2.73 times as likely to develop incident CKD compared to those in the first quartile. Our findings were generally consistent with those reported in three previous cohort studies in mainland China [10, 19, 20]. In a community-based cohort study among 1,410 participants aged 40 years or above, the OR of CKD was 1.19 for each 1 mg/dL increment and 2.14 for the fourth versus first quartile [10], which were equivalent to ours with a similar population-based design. The same study also showed robust associations after excluding participants with hypertension and diabetes. Two other studies in mainland China each using health check-up data for over 5,000 participants from a regional tertiary hospital found 60–70% higher odds of incident CKD for the extreme-quartile comparison of SUA during a follow-up of 5–6 years [19, 20], and one of these two studies showed that the time-mean SUA accounting for fluctuations of SUA could increase the odds of incident CKD by 6.32-fold for the similar comparison [20]. These two hospital-based studies seemed to observe weaker associations than those in general populations, possibly due to a narrower spectrum of participants who might have certain medical conditions, and the much stronger association for time-mean SUA might reflect that average increase of SUA could be a more robust predictor of CKD than the increase of a one-time measurement. Of note, another cohort study among 3,605 participants in Chinese Taiwan showed that progressively elevated SUA and persistently high SUA both were associated with two-fold risk of CKD over a 5-year follow-up, compared to those with persistently lower SUA, which further demonstrates the utility of longitudinal SUA change for predicting incident CKD [21]. In addition, our findings based on both the CKD-EPI and MDRD criteria for determining CKD showed consistent positive associations between SUA and CKD, which agreed well with results from other two Chinese studies using these two sets of criteria [9, 19]. A large retrospective cohort study in over 94,000 Taiwanese participants aged ≥ 20 years showed that participants in the fifth SUA quintile was associated with a 12–15% higher risk of incident CKD compared with those in the first SUA quintile during a mean follow-up of 3.5 years [9]. Furthermore, other large cohort studies in non-Chinese populations in Japan [22], Korea [23], US [24], and Austria [25] supported the positive association between SUA and CKD.

However, null associations were also reported between each 1 mg/dL increment in SUA and incident CKD in a study among 5,888 adults aged ≥ 65 years in the US (OR, 1.00; 95% CI, 0.89–1.14) [26] and another study among 800 adults over 65 years in Chinese Taiwan (1.00; 0.85–1.18) [7], as well as a study in 1,094 employees of an energy generation and distribution company in Brazil (1.12; 0.83–1.50) [27]. These inconsistencies could be potentially explained by age heterogeneity and/or small sample sizes. A previous meta-analysis of 15 cohort studies showed pooled RRs of 1.26 (95% CI, 1.21–1.31) for each 1 mg/dL increment in the < 60 years group versus 1.04 (0.96–1.13) in the ≥ 60 years group (*P* for heterogeneity = 0.004), although the number of studies was small in the older age group [6]. Moreover, while there was a lack of statistical significance for the association between hyperuricemia and incident CKD in our Chinese population, it was suggested otherwise in another meta-analysis of 13 cohort studies [28]. These limitations of and uncertainties around previous original and meta-analysis studies demonstrate the necessity to conduct an additional systematic review to update the current evidence.

Our meta-analysis further extended the evidence beyond the Chinese population. Our meta-analysis of 30 studies among 577,334 participants consistently showed that higher SUA was associated with an increased risk of CKD using SUA as continuous, dichotomous, and categorical quartile variables. Our findings were fairly consistent with two previous meta-analyses published five years ago that contained less than half of the data [6, 28]. Of note, the age heterogeneity for assessing each 1 mg/dL increment of SUA, which existed in a previous meta-analysis [6], was not observed in the current meta-analysis when more data were available. Given the potential link between SUA and CKD and high prevalence of hyperuricemia (up to 8.4% in 2009–2010) in Chinese adults [29], our findings may reinforce the need for timely and routine monitoring of uric acid levels for prevention of incident CKD in clinical practice.

Although the mechanisms underlying the link between SUA and CKD are still uncertain, there have been several lines of evidence. First, uric acid crystals may cause direct damage to the kidney through precipitation in renal tubules [30]. Second, uric acid induces immune system activation and alters the characteristics of resident kidney cells, which contributes to renal inflammation and fibrosis [31]. Third, elevated SUA levels could lead to the development of hypertension that compromises renal function [32]. Fourth, SUA may be a marker of metabolic syndrome and diabetes, which are conventional risk factors for CKD [33]. Fifth, the link between elevated SUA and CKD could be attributable to shared lifestyle risk factors such as unhealthy diet, which could not be fully adjusted for in statistical models.

The current study has major strengths including a large sample size, prospective nature, and a comprehensive meta-analysis. However, several limitations should be acknowledged in our original analyses. First, the study population was part of the CHARLS, and a considerable proportion of participants were excluded due to data missing or loss to follow-up, which may lead to selection and information bias. Second, we could not control for the confounding effects of medications for hyperuricemia such as allopurinol and lifestyle factors such as dietary and physical activity due to data unavailability, and residual confounding was thus likely to exist. Third, the follow-up lasted only 4 years and reverse causality could not be ruled out, i.e., CKD might lead to increased SUA instead of the other way around. However, our meta-analysis consistently confirmed the positive association in subgroups of studies with different follow-up durations. Fourth, we did not have repeated measurements of SUA before CKD ascertainment and could not account for its fluctuation in our analyses. However, our sensitivity analysis using a time-mean measure of SUA supported our overall findings.

## Conclusion

Higher SUA levels were associated with an increased risk of incident CKD in Chinese middle-aged and elderly adults. Such a positive association was supported by findings from a meta-analysis across multiple populations. Given the high prevalence of hyperuricemia in the general population, it is imperative to understand whether reducing SUA levels could lead to lower CKD risk. Future clinical trials and mechanistic studies may improve our understanding of the link between SUA and CKD and inform potential interventions in the population.

## Supplementary Information

**Additional file 1: Table S1.** Comparison of baseline characteristics between participants included and excluded. **Table S2.** Association between SUA levels or hyperuricemia and incident CKD in China using different types of covariates. **Table S3.** Association between SUA levels and incident CKD after multiple imputations of missing data in the CHARLS (N = 5325). **Table S4.** Association between SUA levels and incident CKD in China using the Modification of Diet in Renal Disease (MDRD) study equation for CKD (N = 4489). **Table S5.** Association between time-mean SUA levels and incident CKD in China. **Table S6.** Cohort studies of the relationship between SUA level and risk of incident CKD. **Figure S1.** Flowchart of eligibility of study participants in the CHARLS. **Figure S2.** Association between each 1 mg/dL increment in SUA levels and risk of incident CKD in subgroups in the CHARLS (N = 4546). **Figure S3.** Flowchart of study selection for the meta-analysis.

## Data Availability

The datasets used during the current study are available in the CHARLS official website, http://charls.pku.edu.cn/. All data, analytic methods, and study materials presented within this article are available from the corresponding author on reasonable request.

## Abbreviations

BMI: Body mass index
BP: Blood pressure
CHARLS: China Health and Retirement Longitudinal Study
CKD: Chronic kidney disease
CI: Confidence interval
eGFR: Estimated glomerular filtration rate
FBG: Fasting blood glucose
HbA1c: Glycosylated hemoglobin
HDL: High-density lipoprotein
HR: Hazard ratio
LDL: Low-density lipoprotein
OR: Odds ratio
RR: Relative risk
SD: Standard deviation
SUA: Serum uric acid
TC: Total cholesterol
TG: Triglyceride
