# Supplementary material for: Serum uric acid and risk of incident chronic kidney disease: a national cohort study and updated meta-analysis
Source: Nutr Metab (Lond). 2021 Oct 19;18:94. doi: 10.1186/s12986-021-00618-4 (PMC8524911; doi:10.1186/s12986-021-00618-4)
Supplement: Supplementary file 1 — Additional file 1: Table S1. Comparison of baseline characteristics between participants included and excluded. Table S2. Association between SUA levels or hyperuricemia and incident CKD in China using different types of covariates. Table S3. Association between SUA levels and incident CKD after multiple imputations of missing data in the CHARLS (N = 5325). Table S4. Association between SUA levels and incident CKD in China using the Modification of Diet in Renal Disease (MDRD) study equation for CKD (N = 4489). Table S5. Association between time-mean SUA levels and incident CKD in China. Table S6. Cohort studies of the relationship between SUA level and risk of incident CKD. Figure S1. Flowchart of eligibility of study participants in the CHARLS. Figure S2. Association between each 1 mg/dL increment in SUA levels and risk of incident CKD in subgroups in the CHARLS (N = 4546). Figure S3. Flowchart of study selection for the meta-analysis. [file 12986_2021_618_MOESM1_ESM.docx]

**Additional Appendix**

**Table S1.** Comparison of baseline characteristics between participants included and excluded.

| Characteristics | Total  (N = 7,678) | Study population  ( N = 4,546) | Participants excluded  ( N = 3,132) | *P*-value*** |
| --- | --- | --- | --- | --- |
| Men, n (%) | 3592 (46.8%) | 2099 (46.2%) | 1493 (47.7%) | 0.196 |
| Education level, n (%) |  |  |  | 0.054 |
| Illiterate | 2244 (29.2%) | 1310 (28.8%) | 934 (29.8%) |  |
| Primary school | 3093 (40.3%) | 1882 (41.4%) | 1211 (38.7%) |  |
| Middle school or above | 2340 (30.5%) | 1354 (29.8%) | 986 (31.5%) |  |
| Urban residence, n (%) | 1329 (17.3%) | 626 (13.8%) | 703 (22.4%) | <0.001 |
| Smoking status, n (%) |  |  |  | 0.020 |
| Current | 2345 (30.5%) | 1388 (30.5%) | 957 (30.6%) |  |
| Former | 662 (8.6%) | 359 (7.9%) | 303 (9.7%) |  |
| Never | 4671 (60.8%) | 2799 (61.6%) | 1872 (59.8%) |  |
| Alcohol consumption, n (%) |  |  |  | 0.267 |
| Current | 1985 (25.9%) | 1202 (26.4%) | 783 (25.0%) |  |
| Former | 611 (8.0%) | 368 (8.1%) | 243 (7.8%) |  |
| Never | 5082 (66.2%) | 2976 (65.5%) | 2106 (67.2%) |  |
| Hypertension, n (%) | 2092 (27.2%) | 1171 (25.8%) | 921 (29.4%) | <0.001 |
| Prevalent diabetes mellitus, n (%) | 1224 (15.9%) | 671 (14.8%) | 553 (17.7%) | 0.001 |
| Age, mean (SD), years | 59.17 (9.60) | 58.57 (8.67) | 60.04 (10.74) | <0.001 |
| BMI, mean (SD), kg/m^2^ | 23.41 (3.89) | 23.52 (3.83) | 23.25 (3.98) | 0.002 |

Abbreviations: BMI, body mass index; SUA, serum uric acid; SD, standard deviation.

* *P* value by ANOVA for continuous variables and chi-square test for categorical variables

**Table S2.** Association between SUA levels or hyperuricemia and incident CKD in China using different types of covariates.

|  | OR (95% CI) |
| --- | --- |
| Hyperuricemia vs normal | 1.68 (0.95-2.95) |
| Quartile 4 vs Quartile 1 | 2.57 (1.56-4.22) |
| Each 1 mg/dL increment | 1.46 (1.25-1.70) |

**Abbreviations:** CKD, chronic kidney disease; CI, confidence interval; OR, odds ratio; SUA, serum uric acid.

All model adjusted for age (continuous, years), sex (male and female), residence area (rural and urban), education level (illiterate, primary school, middle school or above), body mass index (continuous, kg/m^2^), smoking status (never, former, and current), and alcohol consumption (never, former, and current), dyslipidemia (yes and no), hypertension (yes and no), and prevalent diabetes mellitus (yes and no).

**Table S3.** Association between SUA levels and incident CKD after multiple imputations for missing data in the CHARLS (N= 5,325).

|  | CKD* / no. of participants | Model 1  OR (95% CI) | Model 2  OR (95% CI) | Model 3  OR (95% CI) |
| --- | --- | --- | --- | --- |
| Hyperuricemia^#^ |  |  |  |  |
| No | 186/5074 | 1.00 (reference) | 1.00 (reference) | 1.00 (reference) |
| Yes | 17/251 | 1.83 (1.09-3.09) | 1.78 (1.05-3.01) | 1.65 (0.97-2.83) |
| SUA levels |  |  |  |  |
| Quartile 1 | 28/1335 | 1.00 | 1.00 | 1.00 |
| Quartile 2 | 43/1333 | 1.54 (0.94-2.50) | 1.52 (0.93-2.47) | 1.50 (0.92-2.45) |
| Quartile 3 | 54/1328 | 2.04 (1.27-3.29) | 2.01 (1.22-3.24) | 1.98 (1.23-3.21) |
| Quartile 4 | 78/1329 | 3.07 (1.93-4.90) | 3.01 (1.88-4.83) | 2.91 (1.80-4.71) |
| *P* for trend |  | *P*<0.001 | *P*<0.001 | *P*<0.001 |
| Each 1 mg/dL |  | 1.50 (1.31-1.71) | 1.49 (1.30-1.72) | 1.48 (1.28-1.71) |

**Abbreviations:** CKD, chronic kidney disease; CI, confidence interval; CHARLS, China Health and Retirement Longitudinal Study; OR, odds ratio; SUA, serum uric acid.

* CKD which was defined as eGFR <60 mL/min/1.73m^2^ based on the Chronic Kidney Disease Epidemiology Collaboration (CKD-EPI).

^#^ Hyperuricemia was defined as a level of SUA ≥7.0 mg/ dL in men or ≥6.0 mg/dL in women.

Model 1: adjusted for age (continuous, years), sex (male and female), residence area (rural and urban), education level (illiterate, primary school, middle school or above). Model 2: adjusted for variables in Model 1 plus body mass index (continuous, kg/m^2^), smoking status (never, former, and current), and alcohol consumption (never, former, and current); Model 3: adjusted for variables in Model 2 plus systolic BP (continuous, mmHg), total cholesterol (continuous, mg/dL), triglyceride (continuous, mg/dL), and prevalent diabetes mellitus (yes and no).

**Table S4.** Association between SUA levels and incident CKD in China using the Modification of Diet in Renal Disease (MDRD) study equation for CKD (N=4,489).

|  | CKD / no. of participants | Model 1  OR (95% CI) | Model 2  OR (95% CI) | Model 3  OR (95% CI) |
| --- | --- | --- | --- | --- |
| Hyperuricemia* |  |  |  |  |
| No | 208/4090 | 1.00 (reference) | 1.00 (reference) | 1.00 (reference) |
| Yes | 16/175 | 1.67 (0.97-2.87) | 1.66 (0.96-2.85) | 1.59 (0.92-2.75) |
| SUA levels |  |  |  |  |
| Quartile 1 | 32/1115 | 1.00 | 1.00 | 1.00 |
| Quartile 2 | 47/1107 | 1.54 (0.97-2.44) | 1.55 (0.98-2.46) | 1.55 (0.98-2.47) |
| Quartile 3 | 65/1133 | 2.36 (1.51-3.67) | 2.37 (1.51-3.70) | 2.36 (1.51-3.70) |
| Quartile 4 | 80/1134 | 3.12 (2.00-4.88) | 3.19 (2.03-5.02) | 3.18 (2.01-5.03) |
| *P* for trend |  | *P*<0.001 | *P*<0.001 | *P*<0.001 |
| Each 1 mg/dL |  | 1.50 (1.31-1.72) | 1.52 (1.32-1.74) | 1.52 (1.32-1.75) |

**Abbreviations:** CKD, chronic kidney disease; CI, confidence interval; OR, odds ratio; SUA, serum uric acid.

* Hyperuricemia was defined as a level of UA ≥7.0 mg/dL in men or ≥6.0 mg/dL in women.

Model 1: adjusted for age (continuous, years), sex (male and female), residence area (rural and urban), education level (illiterate, primary school, middle school or above). Model 2: adjusted for variables in Model 1 plus body mass index (continuous, kg/m^2^), smoking status (never, former, and current), and alcohol consumption (never, former, and current); Model 3: adjusted for variables in Model 2 plus systolic BP (continuous, mmHg), total cholesterol (continuous, mg/dL), triglyceride (continuous, mg/dL), and prevalent diabetes mellitus (yes and no).

**Table S5.** Association between time-mean SUA levels and incident CKD in China*.

|  | CKD^#^ / no. of participants | Model 1  OR (95% CI) | Model 2  OR (95% CI) | Model 3  OR (95% CI) |
| --- | --- | --- | --- | --- |
| UA levels |  |  |  |  |
| Quartile 1 | 25/1137 | 1.00 | 1.00 | 1.00 |
| Quartile 2 | 49/1136 | 1.25 (0.72-2.15) | 1.23 (0.71-2.14) | 1.24 (0.71-2.14) |
| Quartile 3 | 44/1137 | 2.05 (1.23-3.42) | 2.04 (1.22-3.42) | 2.07 (1.23-3.47) |
| Quartile 4 | 82/1136 | 4.11 (2.52-6.71) | 4.16 (2.53-6.86) | 4.16 (2.51-6.90) |
| *P* for trend |  | *P*<0.001 | *P*<0.001 | *P*<0.001 |
| Each 1 mg/dL |  | 1.91 (1.65-2.20) | 1.94 (1.67-2.25) | 1.97 (1.69-2.30) |

**Abbreviations:** CKD, chronic kidney disease; CI, confidence interval; OR, odds ratio; SUA, serum uric acid.

* Time-mean SUA was the average of two measurements of SUA.

^#^ CKD which was defined as eGFR <60 mL/min/1.73m^2^ based on the Chronic Kidney Disease Epidemiology Collaboration.

Model 1: adjusted for age (continuous, years), sex (male and female), residence area (rural and urban), education level (illiterate, primary school, middle school or above). Model 2: adjusted for variables in Model 1 plus body mass index (continuous, kg/m^2^), smoking status (never, former, and current), and alcohol consumption (never, former, and current); Model 3: adjusted for variables in Model 2 plus systolic BP (continuous, mmHg), total cholesterol (continuous, mg/dL), triglyceride (continuous, mg/dL), and prevalent diabetes mellitus (yes and no).

**Table S6**. Cohort studies for the relationship between SUA level and risk of incident CKD.

| **Study (first author, year)** | **Country / region** | **Baseline year** | **Median follow-up (years)** | **Definition of CKD** | **Methods of calculated eGFR** | **Population (mean age)** | **Sample size** | **Incident CKD** | **Type of effect estimate** | **Effect estimate (95% CI)** | **Adjusted covariates** |
| --- | --- | --- | --- | --- | --- | --- | --- | --- | --- | --- | --- |
| Chonchol M, 2007^1^ | US | 1989 | 6.9 | eGFR<60 | MDRD | General population (73.0 years) | 4,610 | 240 | OR | 1.00 (0.89-1.14)* | Age, sex, race, baseline serum creatinine level, BMI, waist circumference, SBP, DBP, use of hypertension medications, use of diuretics, use of allopurinol, glucose level, HDL, TG, ankle-arm index, carotid intima-media thickness, major electrocardiogram  abnormalities, hemoglobin level, CRP, and albumin level |
| Obermayr RP,2008^2^ | Austria | 1990 | 7.0 | eGFR<60 | MDRD | General middle population (Men, 41.6 years; Women 42.2 years) | 17,375 | 288 | OR | 1.69 (1.59-1.80)* | Age, sex, BMI, current-smoker, sports, HDL, hypertension, and DM |
| Weiner DE, 2008^3^ | US | 1987 | 8.5 | eGFR<60 | MDRD | General population (57.4 years) | 12,819 | 741 | OR | 1.07 (1.01-1.14)* | Age, sex, race, DM, blood pressure, cardiac disease, smoke, alcohol use, education, lipid, and albumin |
| Yen CJ, 2009^4^ | Chinese Taiwan | 2002 | 2.7 | eGFR<60 | MDRD | Elderly population (74.5 years) | 800 | 144 | OR | 1.00 (0.85-1.18)* | Age, sex, BMI, DM,  smoking, blood pressure,  hypercholesteraemia,  albuminuria, and serum creatinine |
| Mok Y, 2011^5^ | Korea | 1994 | 10.2 | eGFR<60 | MDRD | General population (44.0 years) | 14,939 | 766 | HR | Men: 1.30 (1.20-1.50)*  Women: 1.10 (1.00-1.20)*  Men: 2.10 (1.60-2.90)^#^  Women: 1.30 (1.00-1.80)^#^ | Age, smoking status, alcohol  drinking, exercise, BMI, TC, hypertension, and DM |
| Yamada T, 2011^6^ | Japan | 2000 | 5.0 | eGFR<60 | Japanese equation | General population (Men, 48.4 years; Women, 49.9 years) | 12,227 | 480 | OR | Men: 1.42 (1.28-1.58)*  Women: 1.32 (1.12-1.56)*  Men: 3.36 (2.24-5.03)^#^  Women: 1.80 (1.14-2.86)^#^ | Age, BMI, elevated blood pressure or hypertension, hypertriglyceridemia, impaired fasting glucose, urinary occult blood, protein, alcohol drinking, and smoking status |
| Ben-Dov IZ,2011^7^ | Israel | 1976 | 26.0 | Hospital discharge diagnosis lists | - | Middle-aged population (Men 50.0 years; Women, 45.0 years) | 2,449 | 109 | HR | Men: 1.94 (1.20-3.14)^#^  Women: 5.20 (1.90-14.20)^#^ | Age, education, glucose, smoking, globulins, TC, stick proteinuria, alcohol, SBP, creatinine, LDL, and DM |
| Wang S, 2011^8^ | Chinese Taiwan | 1996 | 3.5 | eGFR<60 | CKD-EPI | General population (40.7 years) | 94,422 | 3,683 | HR | 1.03 (1.01-1.06)*  Men: 1.03 (1.01-1.06)  Women: 1.03 (0.99-1.08)  1.15 (1.01-1.30)^#^ | Age, sex, education status, drinking status, smoking status, physical exercising, BMI, TG, TC, LDL, HDL, albumin, CRP, g-glutamyl transpeptidase, blood urea nitrogen, hemoglobin, hematocrit, eGFR, proteinuria, hematuria, medical history, family history, and medications. |
| Sonoda H,2011^9^ | Japan | 2001 | 4.6 | eGFR<60 | Japanese equation | General population (52.8 years) | 7,078 | 568 | OR | 1.09 (1.01-1.18)* | BMI, SBP, FPG, LDL, HDL, smoke, hemoglobin, and eGFR |
| Kawashima M, 2011^10^ | Japan | 1990 | 7.9 | eGFR<60 | Japanese equation | Male factory workers (43.0 years) | 1,285 | 100 | HR | 3.99 (2.59-6.15)^＆^ | Age, BMI, HDL, blood pressure, and fasting blood sugar |
| Zhang L, 2012^11^ | China | NA | 4.0 | eGFR<60 | Chinese euqation | General population (59.1 years) | 1,410 | 168 | OR | 1.19 (1.04-1.38)*  2.14 (1.22-3.75)^#^ | Age, sex, BMI, current smoking, hypertension, diabetes, albuminuria, and baseline eGFR |
| Sedaghat S, 2013^12^ | Netherlands | 1990 | 6.5 | eGFR<60 | MDRD | General population (70.4 years) | 2,154 | 249 | HR | 1.12 (0.98-1.28)* | Age, sex, SBP, BMI, alcohol consumption, smoking, HDL, DM, coronary heart disease, TC, diuretics, beta blockers, calcium channel blockers, ACE inhibitors, and baseline eGFR |
| Chang HY,2013^13^ | Chinese Taiwan | 2008 | 4.0 | ACR>30 | - | Middle-aged and elderly population (66.1 years) | 993 | NA | HR | 1.42 (1.27-1.59)*  Men: 1.21 (1.02-1.44)  Women: 1.57 (1.35-1.82)  3.54 (2.11-5.93)^#^  Men: 4.58 (1.50-14.03)  Women: 3.17 (1.55-6.48) | sex, age, DM, hypertension, TG, TC, eGFR, and BMI |
| Ryoo JH, 2013^14^ | Korea | 2005 | 4.0 | eGFR<60 | CKD-EPI | Health man (mean 41.8 years) | 18,778 | 110 | OR | 3.03 (1.48-6.19)^#^ 1.96 (1.28-2.99)^＆^ | Age, baseline eGFR, SBP, HOMA-IR, TG , BMI, alcohol intake, smoking status, regular exercise, hypertension, and DM |
| Kamei K, 2014^15^ | Japan | 2008 | 2.0 | eGFR<60 | Japanese equation | General population (63.3 years) | 141,514 | 9,169 | OR | 1.06 (1.04-1.08)*  Men: 1.06 (1.02-1.09)  Women: 1.08 (1.04-1.11)  1.20 (1.12-1.30) ^#^  Men: 1.24 (1.11-1.40)  Women: 1.22 (1.10-1.35)  1.12(1.05-1.19)^＆^  Men: 1.13 (1.05-1.23)  Women: 1.14 (1.04-1.25) | Age, sex, obesity, hypertension, DM, dyslipidemia, smoking, alcohol consumption, eGFR, and proteinuria |
| Kuriyama S, 2014^16^ | Japan | 2008 | 4.0 | eGFR<60 | Japanese equation | Office workers (39.0 years) | 8,223 | 139 | OR | 1.08 (0.91-1.29)* | Age, sex, eGFR, SBP , DBP, TC, TG, HbA1c, and BMI |
| Toda A, 2014^17^ | Japan | 2002 | 5.0 | eGFR<60 | Japanese equation | General population (52.1 years) | 1,652 | 93 | HR | 1.36 (1.10-1.66)* | Age, sex, BMI, SDP, DBP, HDL, LDL, TG, HbA1c, and HOMA-IR |
| Chou YC, 2014^18^ | Chinese Taiwan | 2002 | 5.2 | eGFR<60 | CKD-EPI | General population (41.2 years) | 3,605 | 233 | HR | 1.90 (1.34-2.71)^#^ | Sex, age, hypertension status, BMI, TC , TG,  FPG level, and eGFR |
| Toyama T,2015^19^ | Japan | 1998 | 4.0 | eGFR<60 | NA | General population (45.4 years) | 41,632 | 3,186 | HR | 1.23 (1.08-1.41)^#^ | Age, eGFR, SBP, BMI, hemoglobin, HDL, TG, urinary protein, and DM |
| Takae K, 2016^20^ | Japan | 2002 | 5.0 | ACR>30 and eGFR<60 | Japanese equation | General population (59.0 years) | 2,059 | 396 | OR | 1.18 (1.05-1.32)*  2.10 (1.37-3.23)^#^ | Age, sex, SBP, the use of antihypertensive agents, DM, TC, HDL, BMI, hemoglobin, use of UA-lowering agents, log hs-CRP, eGFR, log U-ACR, smoking habit, alcohol intake, and regular exercise |
| Chini LSN, 2017^21^ | Brazil | 2008 | 5.1 | eGFR<60 | CKD-EPI | Employees of an energy generation and distribution company (48.7 years) | 1,094 | 44 | OR | 1.12 (0.83-1.50)* | Age, sex, DM, hypertension, HDL, TG, sedentary lifestyle, BMI, and smoking |
| Mwasongwe SE, 2018^22^ | US | 2000 | 8.1 | eGFR<60 | CKD-EPI | General population (55.3 years) | 3,556 | 268 | OR | 2.00 (1.31-3.06)^#^  Men: 1.81 (0.89-3.68)  Women: 1.96 (1.11-3.46) | Age, sex, BMI, eGFR, gout medications, loop diuretics, thiazide diuretics, potassium-sparing diuretics, antihyperlipidemics, DM, TC, CRP, and U-ACR |
| Ye M, 2018^23^ | China | 2011 | 6.0 | eGFR<60 | NA | General population (48.0 years) | 5,183 | 227 | OR | 1.69 (1.06-2.70)^#^ | Age, sex, BMI, SBP, DBP, TC, baseline eGFR, FPG, hyperuricemia, hypertension, and DM |
| Cao X, 2018^24^ | China | 2011 | 4.4 | eGFR<60 | CKD-EPI | Health population (49.0 years) | 6,495 | 372 | OR | 1.63 (1.02-2.97)^#^ | Age, BMI, DM, hypertension, alcohol drinking, SBP, TC,  and eGFR |
| Mun KH, 2018^25^ | Korea | 2005 | 3.9 | eGFR<60 | CKD-EPI | General population (Men, 61.5 years; Women, 59.6 years) | 5,577 | 580 | HR | Men: 1.60 (1.02-2.51)^#^  Women: 1.56 (1.14-2.15)^#^ | Age, eGFR, smoking, alcohol, exercise, marriage, education, hypertension, DM, BMI, glucose, triacylglycerol levels, TC, and HDL |
| Nakayama S, 2021^26^ | Japan | 2008 | 4.7 | eGFR <60 or proteinuria | modified Japanese equation | General population  (44.1 years) | 138,511 | 12,589 | HR | Men: 3.74 (1.68–8.35)^#^  Women: 3.20 (0.80–12.8)^#^ | Age, BMI, current or ex-smoker, current or ex-drinker, diabetes mellitus, dyslipidemia, SBP, antihyperuricemic drug usage, and baseline eGFR |
| Tada K, 2021^27^ | Japan | 2008 | 4.6 | eGFR <60 | NA | General population  (60.4 years) | 5,507 | 757 | HR | 1.13 (1.03–1.24)*  1.49 (1.13–1.95)^#^ | Sex, age, smoking, obesity, hypertension, diabetes,  and dyslipidemia |
| Son YB, 2021^28^ | Korea | 2004 | 3.0 | eGFR <60 | NA | General population  (44.5 years) | 13,133 | 101 | RR | 2.22 (1.18–4.18)^#^ | Age, sex, SBP, BMI, eGFR, hemoglobin, white blood cell, LDL, aspartate aminotransferase, alanine aminotransferase, fasting blood sugar, serum albumin, TC and malignancy |
| Chang PY, 2021^29^ | Chinese Taiwan | 2008 | 8.0 | eGFR <60 | CKD-EPI | General population  (64.5 years) | 3,708 | NA | RR | Men: 0.95 (0.85–1.05)*  Women: 1.11 (1.01–1.22)* | Age, CKD,  hypertension, diabetes mellitus, dyslipidemia, gout, stroke, BMI, serum creatinine, cigarette smoking, and alcohol consumption |
| Current study | China | 2011 | 4.0 | eGFR<60 | CKD-EPI | General population (58.6 years) | 4,546 | 180 | OR | 1.49 (1.28-1.74)*  2.73 (1.65-4.50)^#^  1.73 (0.98-3.06)^＆^ | Age, sex, residence area, education level, smoking status, alcohol consumption, BMI, SBP, TC, TG, and DM |

Abbreviations: BMI, body mass index; CI, confidence interval; CKD, chronic kidney disease; CKD-EPI, Chronic Kidney Disease Epidemiology Collaboration; CRP, C-reactive protein; DBP, diastolic blood pressure; DM, diabetes mellitus; eGFR, estimated glomerular filtration rate; FPG, fasting plasma glucose; hs-CRP, high-sensitivity C-reactive protein; HR, hazard ratio; HOMA-IR, homeostasis model assessment insulin resistance; HbA1c, glycated hemoglobin; HDL, high density lipoprotein; LDL, low density lipoprotein; MDRD, Modification of Diet in Renal Disease; NA, not available; OR, odds ratios; SBP, systolic blood pressure; SUA, serum uric acid; TC, total cholesterol; TG, triglycerides; UA, uric acid; U-ACR, urine albumin-creatinine ratio.

* Effect estimate calculated for each 1 mg/dL increment in SUA in relation to incident CKD.

^#^ Effect estimate calculated for comparing the highest SUA quartile versus the lowest SUA quartile in relation to incident CKD.

^＆^ Effect estimate calculated for SUA as a binary variable (hyperuricemia versus no hyperuricemia) in relation to incident CKD.

**Figure S1.** Flowchart of eligibility of study participants in the CHARLS. CKD-EPI-based outcomes: means that glomerular filtration rate (GFR) was calculated with CKD-EPI equation. **Abbreviations:** BMI, body mass index; BP, blood pressure; CHARLS, China Health and Retirement Longitudinal Study; CKD-EPI, Chronic Kidney Disease Epidemiology Collaboration; eGFR, estimated glomerular filtration rate.


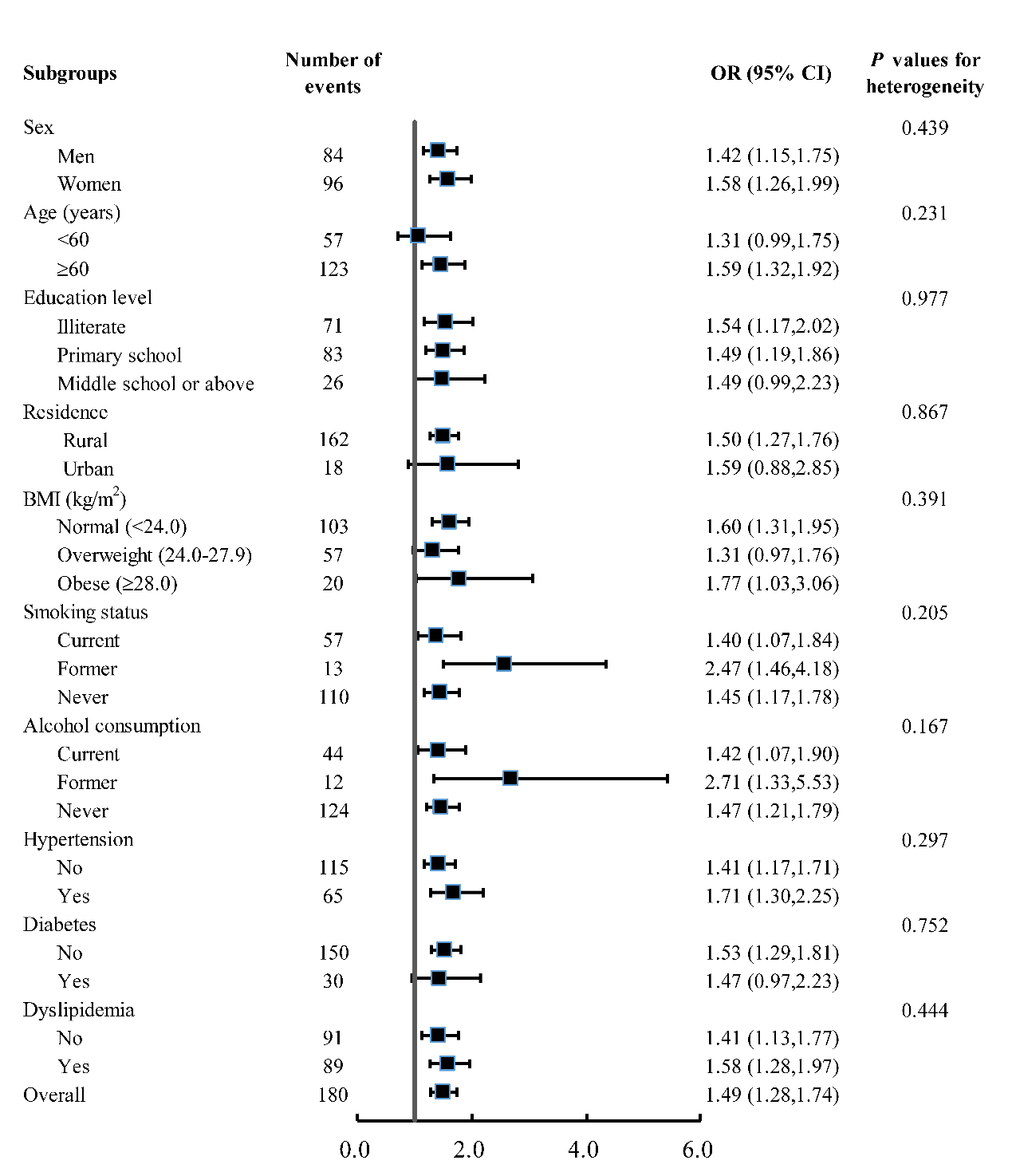


**Figure S2.** Association between each 1 mg/dL increment in SUA levels and risk of incident CKD in subgroups in the CHARLS (N=4,546). **Abbreviations:** BMI, body mass index; CKD, chronic kidney disease; CHARLS, China Health and Retirement Longitudinal Study; CI, confidence interval; OR, odds ratio; SUA, serum uric acid.

Models were adjusted for age (continuous, years), sex (male and female), residence area (rural and urban), education level (illiterate, primary school, middle school or above), body mass index (continuous, kg/m^2^), smoking status (never, former, and current), and alcohol consumption (never, former, and current), systolic BP (continuous, mmHg), total cholesterol (continuous, mg/dL), triglyceride (continuous, mg/dL), and prevalent diabetes mellitus (yes and no).

MEDLINE

(N = 1,934)

EMBASE

(N = 4,201)

Full-text articles assessed (N = 64)

Records identified through database searching (N = 6,135)

Records after excluding non-cohort studies (N = 1,300)

Records excluded based on the title and abstract:

1. Duplicate studies (n = 87)

2. Review and meta-analysis (n = 40)

3. Irrelevant studies (n = 1,109)

35 full-text articles excluded:

1. Irrelevant studies (n = 15)

2. Studies in children (n = 10)

3. Studies with insufficient data (n = 4)

4. Randomized controlled trials (n = 1)

5. Conference abstracts (n = 5)

Included in the meta-analysis (N = 30, including the current study)

**Figure S3.** Flowchart of study selection for the meta-analysis.

References

1. Chonchol M, Shlipak MG, Katz R, et al. Relationship of uric acid with progression of kidney disease. *Am J Kidney Dis.* 2007; **50**: 239-247.

2. Obermayr RP, Temml C, Knechtelsdorfer M, et al. Predictors of new-onset decline in kidney function in a general middle-european population: Nephrology Dialysis Transplantation. *Nephrol Dial Transplant.* 2008; **23**: 1265-1273.

3. Weiner DE, Tighiouart H, Elsayed EF, et al. Uric acid and incident kidney disease in the community. *J Am Soc Nephrol.* 2008; **19**: 1204-1211.

4. Yen CJ, Chiang CK, Ho LC, et al. Hyperuricemia Associated With Rapid Renal Function Decline in Elderly Taiwanese Subjects. *J Formos Med Assoc.* 2009; **108**: 921-928.

5. Mok Y, Lee SJ, Kim MS, et al. Serum uric acid and chronic kidney disease: the Severance cohort study. *Nephrol Dial Transplant.* 2012; **27**: 1831-1835.

6. Yamada T, Fukatsu M, Suzuki S, et al. Elevated serum uric acid predicts chronic kidney disease. *Am J Med Sci.* 2011; **342**: 461-466.

7. Ben-Dov IZ, Kark JD. Serum uric acid is a GFR-independent long-term predictor of acute and chronic renal insufficiency: the Jerusalem Lipid Research Clinic cohort study. *Nephrol Dial Transplant.* 2011; **26**: 2558-2566.

8. Wang S, Shu Z, Tao Q, et al. Uric acid and incident chronic kidney disease in a large health check-up population in Taiwan. *Nephrology (Carlton).* 2011; **16**: 767-776.

9. Sonoda H, Takase H, Dohi Y, et al. Uric acid levels predict future development of chronic kidney disease. *Am J Nephrol.* 2011; **33**: 352-357.

10. Kawashima M, Wada K, Ohta H, et al. Association between asymptomatic hyperuricemia and new-onset chronic kidney disease in Japanese male workers: a long-term retrospective cohort study. *BMC Nephrol.* 2011; **12**: 31.

11. Zhang L, Wang F, Wang X, et al. The association between plasma uric acid and renal function decline in a Chinese population-based cohort. *Nephrol Dial Transplant.* 2012; **27**: 1836-1839.

12. Sedaghat S, Hoorn EJ, Van Rooij FJA, et al. Serum uric acid and chronic kidney disease: The role of hypertension. *PLoS One*. 2013; **8**: e76827

13. Chang HY, Lee PH, Lei CC, et al. Hyperuricemia is an independent risk factor for new onset micro-albuminuria in a middle-aged and elderly population: a prospective cohort study in taiwan. *PLoS One.* 2013; **8**: e61450.

14. Ryoo JH, Choi JM, Oh CM, et al. The association between uric acid and chronic kidney disease in Korean men: a 4-year follow-up study. *J Korean Med Sci.* 2013; **28**: 855–860.

15. Kamei K, Konta T, Hirayama A, et al. A slight increase within the normal range of serum uric acid and the decline in renal function: associations in a community-based population. *Nephrol Dial Transplant.* 2014; **29**: 2286-2292.

16. Kuriyama S, Maruyama Y, Nishio S, et al. Serum uric acid and the incidence of CKD and hypertension. *Clin Exp Nephrol.* 2015; **19**: 1127-1134.

17. Toda A, Ishizaka Y, Tani M, et al. Hyperuricemia is a significant risk factor for the onset of chronic kidney disease. *Nephron Clin Pract.* 2014; **126**: 33-38.

18. Chou YC, Kuan JC, Yang T, et al. Elevated uric acid level as a significant predictor of chronic kidney disease: a cohort study with repeated measurements. *J Nephrol.* 2015; **28**: 457-462.

19. Toyama T, Furuichi K, Shimizu M, et al. Relationship between Serum Uric Acid Levels and Chronic Kidney Disease in a Japanese Cohort with Normal or Mildly Reduced Kidney Function. *PLoS One.* 2015; **10**: e0137449.

20. Takae K, Nagata M, Hata J, et al. Serum Uric Acid as a Risk Factor for Chronic Kidney Disease in a Japanese Community - The Hisayama Study. *Circ J.* 2016; **80**: 1857-1862.

21. Chini LSN, Assis LIS, Lugon JR. Relationship between uric acid levels and risk of chronic kidney disease in a retrospective cohort of Brazilian workers. *Braz J Med Biol Res.* 2017; **50**: e6048.

22. Mwasongwe SE, Fulop T, Katz R, et al. Relation of uric acid level to rapid kidney function decline and development of kidney disease: The Jackson Heart Study. *J Clin Hypertens (Greenwich).* 2018; **20**: 775-783.

23. Ye M, Hu K, Jin J, et al. The association between time-mean serum uric acid levels and the incidence of chronic kidney disease in the general population: a retrospective study. *BMC Nephrol.* 2018; **19**: 190.

24. Cao X, Wu L, Chen Z. The association between elevated serum uric acid level and an increased risk of renal function decline in a health checkup cohort in China. *Int Urol Nephrol.* 2018; **50**: 517-525.

25. Mun KH, Yu GI, Choi BY, et al. Effect of Uric Acid on the Development of Chronic Kidney Disease: The Korean Multi-Rural Communities Cohort Study. *J Prev Med Public Health.* 2018; **51**: 248-256.

26. Nakayama S, Satoh M, Tatsumi Y, et al. Detailed association between serum uric acid levels and the incidence of chronic kidney disease stratified by sex in middle-aged adults. *Atherosclerosis.* 2021;S0021-9150(21)01193-X.

27. Tada K, Maeda T, Takahashi K, et al. Association between serum uric acid and new onset and progression of chronic kidney disease in a Japanese general population: Iki epidemiological study of atherosclerosis and chronic kidney disease. *Clin Exp Nephrol.* 2021;**25**:751-759

28. Son YB, Yang JH, Kim MG, Jo SK, Cho WY, Oh SW. The effect of baseline serum uric acid on chronic kidney disease in normotensive, normoglycemic, and non-obese individuals: A health checkup cohort study. *PLoS One.* 2021;**16**:e0244106.

29. Chang PY, Chang YW, Lin YF, Fan HC. Sex-Specific Association of Uric Acid and Kidney Function Decline in Taiwan. *J Pers Med.* 2021;**11**:415.
